# 3D Super-Resolution Optical Profiling Using Microsphere Enhanced Mirau Interferometry

**DOI:** 10.1038/s41598-017-03830-6

**Published:** 2017-06-16

**Authors:** Ivan Kassamakov, Sylvain Lecler, Anton Nolvi, Audrey Leong-Hoï, Paul Montgomery, Edward Hæggström

**Affiliations:** 1 University of Helsinki, Helsinki, Finland; 2 ICube Laboratory, University of Strasbourg-CNRS, Strasbourg, France

## Abstract

We present quantitative three dimensional images of grooves on a writable Blu-ray Disc based on a single objective Mirau type interferometric microscope, enhanced with a microsphere which is considered as a photonic nanojet source. Along the optical axis the resolution of this microsphere assisted interferometry system is a few nanometers while the lateral resolution is around 112 nm. To understand the physical phenomena involved in this kind of imaging we have modelled the interaction between the photonic jet and the complex disc surface. Agreement between simulation and experimental results is demonstrated. We underline that although the ability of the microsphere to generate a photonic nanojet does not alone explain the resolution of the interferometer, the nanojet can be used to try to understand the imaging process. To partly explain the lateral super-resolution, the potential role of coherence is illustrated. The presented modality may have a large impact on many fields from bio-medicine to nanotechnology.

## Introduction

Bio-imaging, especially label-free bio-imaging, is globally large and rapidly growing both academically and commercially. Primary trends are three dimensional (3D) imaging, super-resolution imaging (Nobel Prize for Chemistry 2014)^1^ label-free imaging (no fluorophores, dyes or nanomarkers), quantitative (metrologically traceable) imaging, high-throughput imaging (currently hundreds of samples per microscope per day), and fresh sample imaging (little or no sample preparation). We address the first three issues. Our approach is to use a single objective Mirau type interferometric microscope enhanced with a microsphere that is considered as a photonic nanojet lens capable of achieving super-resolved and quantified 3D label-free imaging.

The wave nature of light limits the 3D resolution in classical optical microscopy^2, 3^. The diffraction limit applies to far-field imaging where the specimen is many thousands of wavelengths from the objective. Super-resolution techniques create images with higher resolution than the Abbe limit^4^. Many such modalities exist^5–7^ but these techniques require labelling agents. Near-field optical techniques are label-free, but they are limited to surface and near surface imaging^8^. One of the few 3D label-free techniques is tomographic diffractive microscopy that achieves super-resolution using a synthetic numerical aperture^9^, giving a typical resolution limited to λ/4.

Interference microscopy, including phase shifting microscopy and coherence scanning interferometry (a.k.a scanning white light interferometry, SWLI), is a mature non-contact technique that provides quantitative information across large areas with sub-nanometer axial resolution^2, 10^. The technique is able to resolve and measure closely spaced 3D details such as those found in nanometric surface roughness and even atomic step heights as long as the lateral details of the features can be resolved. But since the method is diffraction limited, this means that features laterally smaller than the diffraction limit cannot be measured. The ability to overcome this limitation is demonstrated in this paper using a single objective Mirau type interferometric microscope enhanced with a photonic nanojet lens.

A photonic nanojet is a narrow light beam situated near the shadow-side surface of an illuminated dielectric microsphere whose diameter is a few wavelengths of the light source^11, 12^. The nanojet exhibits a high intensity (100x the incident power density), a sub-diffraction beam width and an axial length of several wavelengths. We show how the ability of the microsphere to generate a photonic nanojet can be used to try to understand the imaging presented in this paper and how the coherence can play a role in partly explaining the super-resolution.

## Results

Recently, lateral super-resolution has been achieved using nanolenses, microdroplets, and microspheres placed on the sample below the microscope objective^13^. Silica (n = 1.46) with Ø = 2–9 µm, and barium titanate microspheres (n = 1.9) with Ø = 100 µm have provided images of recordable Blu-ray Disc line structures, nanopores, and adenoviruses^14, 15^. The lateral resolution of these systems was less than 100 nm. It is unclear exactly how this microsphere approach works^13–15^ since the sub-diffraction width (around λ/3) of the photonic nanojet cannot by itself explain super-resolution. One of the conditions necessary to achieve super-resolution is to look “through the microsphere” at a virtual image produced by the microsphere^13^. By considering the microsphere as a photonic jet lens, the imaging properties can be investigated numerically and the existence of the virtual plane justified. We present such a study in the following section.

### Simulations

**Figure 1:**
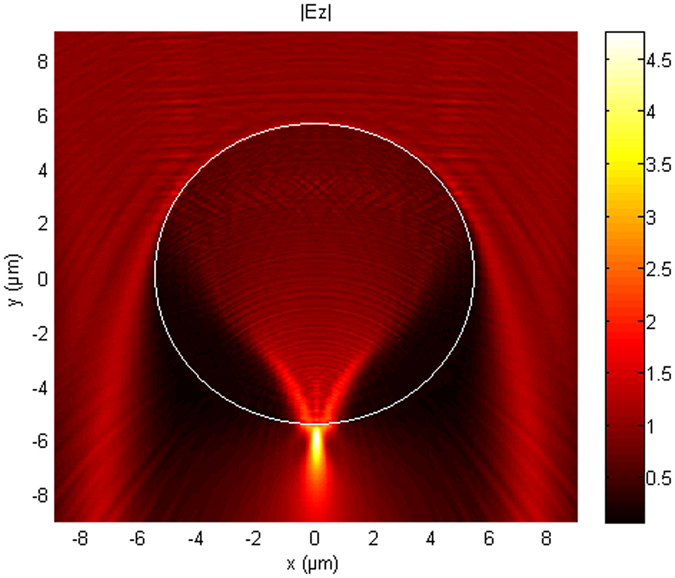
The formation of a photonic nanojet resulting from unitary (1 V/m) plane waves (λ from 400 nm to 1000 nm) illuminating (from the top) an 11 µm microsphere (n = 1.68) using 2D FEM (TE wave case) electric field simulation (absolute values |E_z_|).

While light is scattered by a microsphere in the far field, it can be concentrated in its vicinity in the so-called photonic nanojet. By analogy, this concentration can be considered as being at the focus of the microsphere. The simulation, created using finite element method (FEM), of a photonic jet created by a microsphere (Ø = 11 µm, n = 1.68) illuminated by a white light incident plane wave is shown in Fig. 1. The photonic nanojet is 2 µm long (full width at half maximum (FWHM) along the y axis) and 0.5 µm wide (FWHM along the x axis). If the microsphere is considered as a classical lens, the photonic jet would be its point spread function and the expected resolution would be 0.5 µm. The maximum intensity is 0.5 µm below the microsphere along the y axis. By analogy with an imaging lens, since the nanojet is outside the microsphere, an object placed between the microsphere and the photonic jet may be magnified in a virtual image plane. Beyond this analogy the studies of the photonic nanojet have shown that the microspheres do not obey the laws of geometrical optics and that rigorous electromagnetic methods are required^11, 12^.

**Figure 2:**
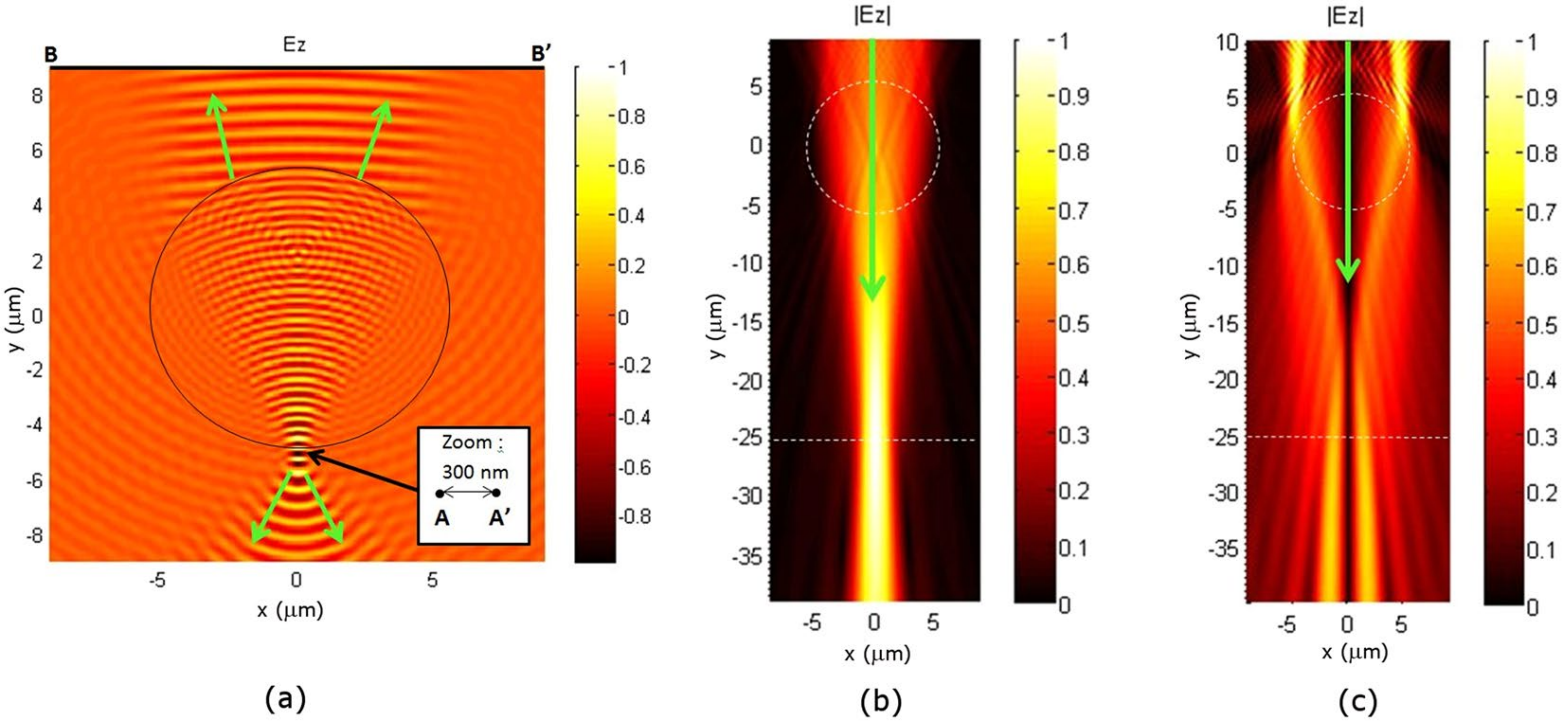
Attempts to predict the performance of the microsphere assisted interferometric microscope. The virtual image plane positions (dashed lines) are determined using 2D FEM (TE wave case) simulations for an 11 µm microsphere (n = 1.68) and light at a wavelength of λ = 600 nm. The arrows indicate the direction of light propagation. (**a**) Electric field (real part) propagation from two point sources A and A’ that are in phase, separated by 300 nm, and positioned below the microsphere in the plane at y = −5.5 µm. (**b**) Time-reversal propagation, without the sphere, of the wavefront emanating from [B, B’] in (**a**) (absolute values |E_z_|). The virtual image plane is 20 µm below the microsphere. (**c**) As in (**b**) when the two sources A and A’ in (**a**) are out of phase.

We have investigated the “virtual plane” hypothesis. To determine the position of this virtual image plane, we first simulated two point sources A and A’ placed in the sample plane, separated by 300 nm and emitting at a wavelength of λ = 600 nm (Fig. 2a). The resulting wavefront emerging from the top of the microsphere (Ø = 11 µm, n = 1.68) is curved. Since the centre of this wavefront gives the approximate position of the virtual image plane, this plane can now be found by time-reversal propagation of the wavefront from [B, B’] (Fig. 2a), without the microsphere.

The results when A and A’ are in phase, are shown in Fig. 2b where the dashed sphere indicates the initial position of the sphere. The virtual images of the two point sources appear as overlapping diffraction patterns situated at the maximum of the central intensity along *y* (dashed line at *y* = −25 µm in Fig. 2b). The depth of field extends 5 µm to either side of the point of maximum intensity. While the virtual image position is found, justifying the photonic jet lens concept, the two point sources are not resolved.

One hypothesis for explaining super-resolution, yet to be confirmed, is that the microsphere converts the high frequency spatial components of the evanescent field into propagating modes that allow the far-field imaging of sub-diffraction-limited sample features^15^. In addition to this idea, the role of the coherence of the illumination should be accounted for when trying to explain the physics of microsphere-assisted super-resolution^16^. We have also investigated this latter hypothesis by repeating the simulations with the two point sources A and A’ being out of phase. The virtual image, that can be identified by the diffraction patterns from the two point sources can be distinguished at the same position (dashed line at *y* = −25 µm in Fig. 2c). The two intensity maxima are now separated along *x* by 2 µm and the two points are resolved, corresponding to a lateral magnification of x6 (2 µm/0.300 µm). This result indicates that the phase may be involved in super-resolution. Thus, microsphere-assisted imaging could provide lateral super-resolution and the phase could concurrently be exploited to quantify the third dimension, leading to microsphere-assisted interferometry.

### Experimental setup

**Figure 3:**
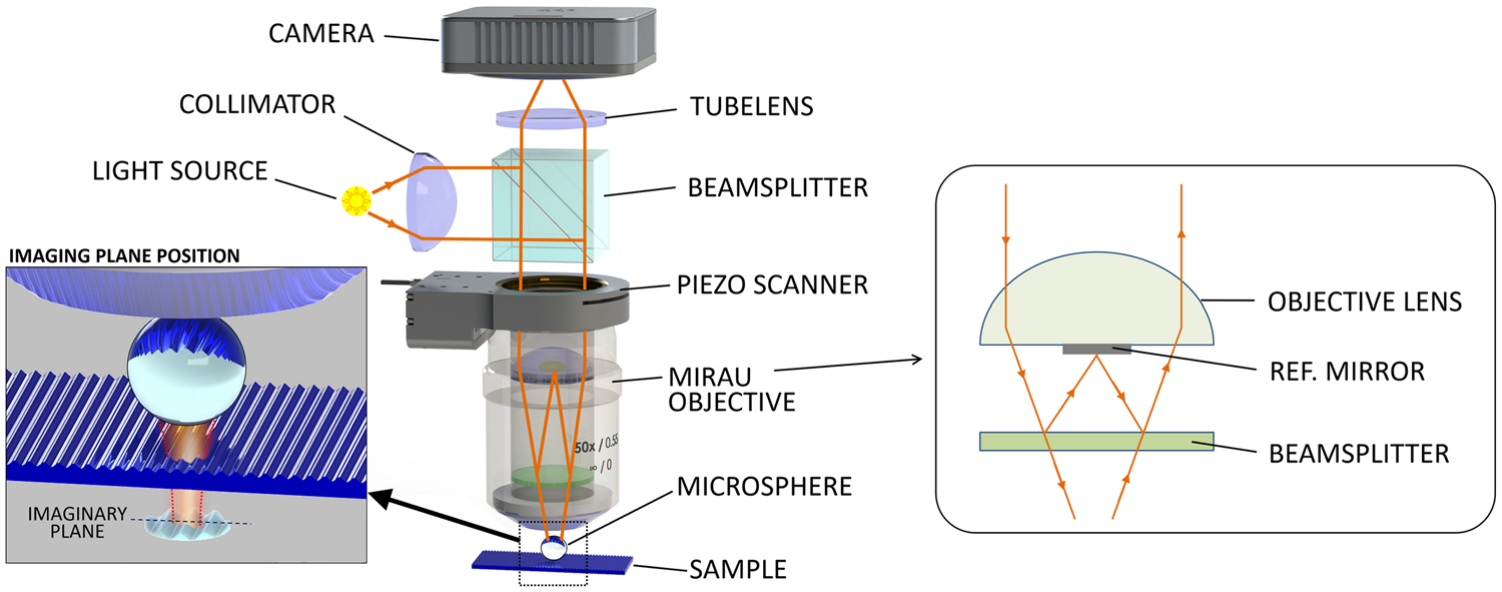
Microsphere-assisted imaging: setup with a Mirau type interferometer and a microsphere placed on top of an object. The sphere projects a virtual image into the far-field that is collected by the objective lens. Zoom-in is not to scale. The insert on the right shows the design and the ray propagation in a single objective Mirau type interferometer.

We designed an experiment to show that microsphere-assisted interference microscopy provides label-free nano-3D imaging^17^. To increase the lateral resolution of our custom-built SWLI system we placed a drop of water suspended polymer spheres (melamine formaldehyde, Corpuscular Inc., Ø = 11 µm, n = 1.68) on the Blu-ray Disc (Verbatim BD-R, Datalife)^18, 19^ surface and left it to dry. The colloidal spheres form an ordered monolayer through self-assembly^13^. The single lens system uses a Mirau-type interferometric objective (Nikon 50x, *NA* = 0.55) with white light illumination (halogen lamp - Philips 7724, 12 V, 100 W) centred at λ = 600 nm, which under normal conditions gives a classical lateral resolution of 0.67 µm. The images were acquired using a high-sensitivity, small pixel size, black and white CMOS camera (Hamamatsu ORCA-Flash 2.8) (Fig. 3).

The surface features of a recordable Blu-ray Disc whose protective polymer layer had been removed were also measured by a metrological AFM system^20^ and by SEM (Hitachi S-4800 FESEM) with a lateral resolution of 2.5 nm^21^ and calibrated using a SIRA SEM calibration specimen S170^22^. For SEM imaging, the disc surface was covered with a 4 nm thick gold layer and care was taken to limit the electron beam power to avoid altering the disc surface.

### Simulation and experimental results

**Figure 4:**
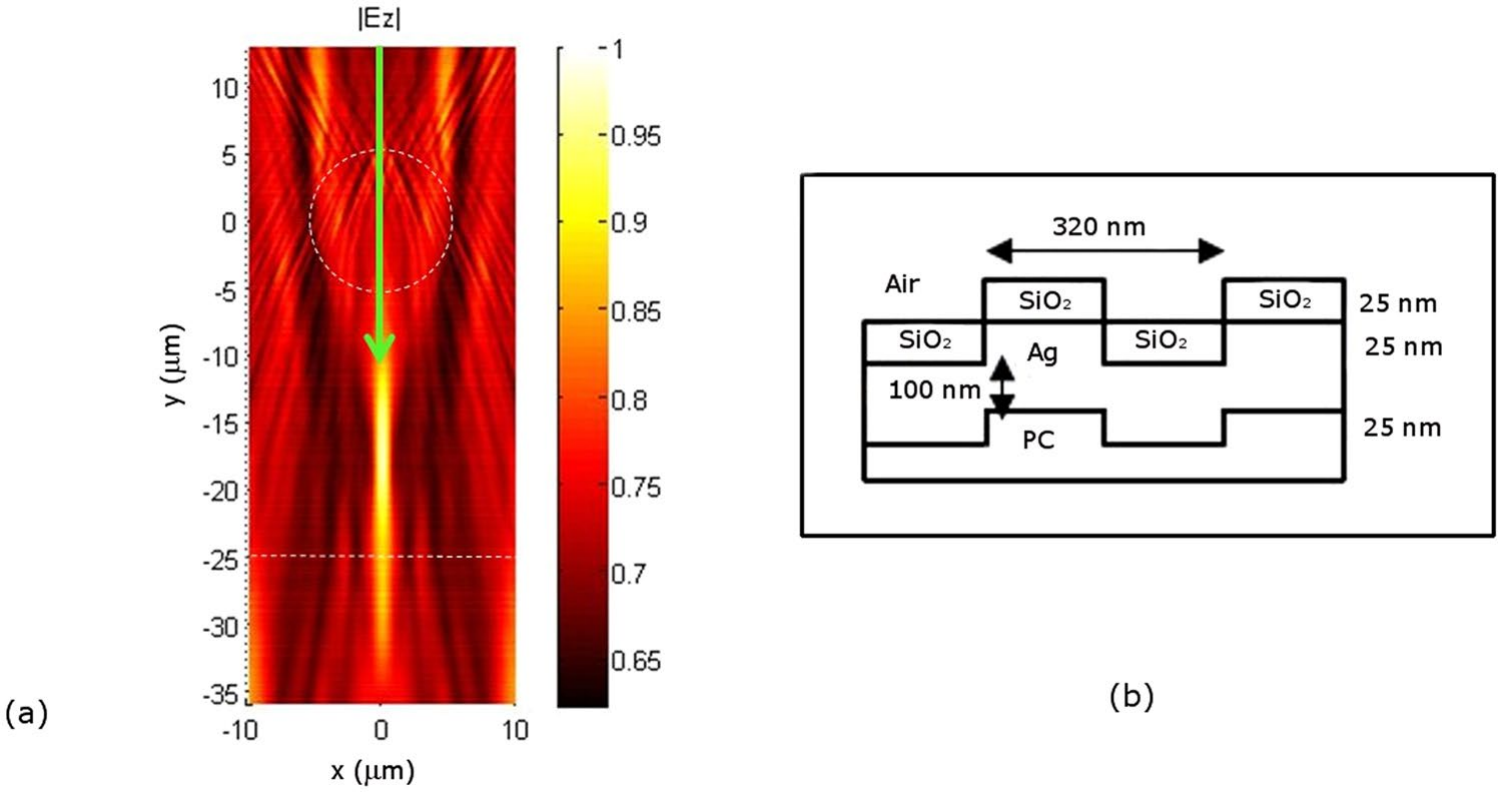
Simulation of instrument interaction with the Blue-ray Disc (**a**). Determining the position of the virtual image (dashed line) plane using 2D FEM simulation, absolute electric field |E_z_|. The first interaction (not represented) of a plane wave with the 11 µm sphere and the Blu-ray Disc (see (**b**) for the Blu-ray Disc model) allows the calculation of the outgoing wave which is then time-reversed and propagated without the sphere. The arrow indicates the direction of light propagation.

To predict the expected super-resolved image we repeated the simulation procedure described previously for the entire spectral range of the instrument. The two point sources were replaced by the Blu-ray Disc, illuminated by a plane wave passing through the Ø = 11 µm microsphere (Fig. 4a). The disc is a complex structure^23^. We approximated its surface by a multilayer rectangular grating with a 320 nm period made of a polycarbonate substrate (n_PC_ = 1.55), a 100 nm thick silver reflective layer, and a 25 nm thick SiO_2_ dielectric layer (n_SiO2_ = 1.5), as shown in Fig. 4b. The light dispersion by the silver was taken into account^24^. For the time-reversal propagation of the wave reflected by the sample (Fig. 4a), although the localization of the virtual image plane is not so obvious since several maxima are visible, the plane can be seen to be at the position predicted in Fig. 2b,c, 20 µm below the sphere (plane at y = −25 µm).

**Figure 5:**
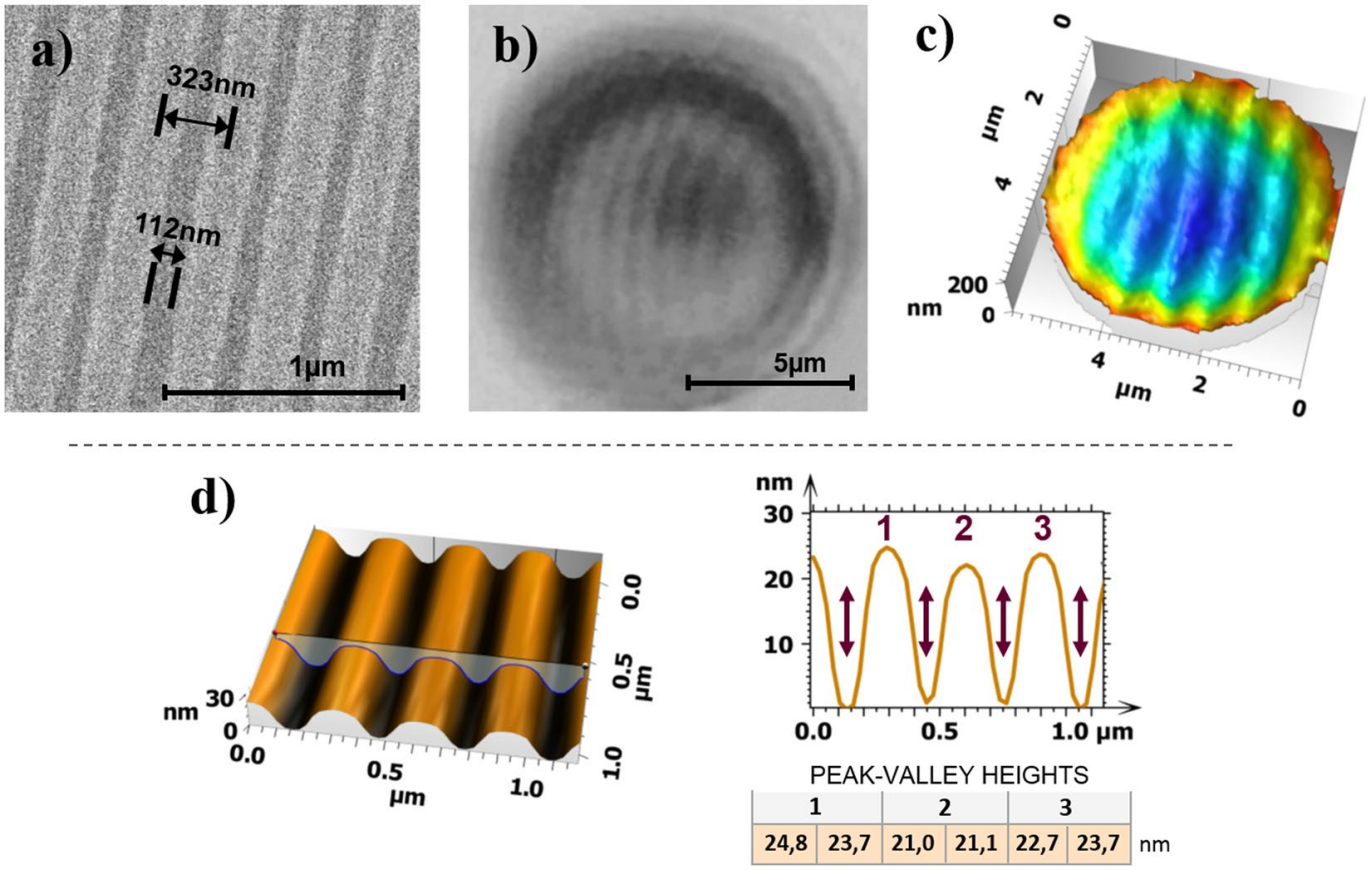
Blu-ray Disc: (**a**) SEM image and a groove measurement. (**b**) 2D image through microsphere. (**c**) Uncorrected 3D image with aberrations measured by microsphere assisted interferometry. (**d**) AFM 3D and cross sectional profile showing resolved grooves 21.0–24.8 nm deep.

The experimental measurements partly validate the simulation predictions. From the SEM image (Fig. 5a) the inter groove distance is found to be 323 ± 2.5 nm and the width 112 ± 2.5 nm. Figure 5b,c show the results of a microsphere-assisted interferometry measurement of the Blu-ray Disc surface using the previously described system. The 2D image through the sphere is shown in Fig. 5b, while the non-corrected 3D image with aberrations is depicted in Fig. 5c. A 3D structure is clearly observed and the grooves resolved. This demonstrates a near 6-fold increase in lateral resolution compared to measurements made with the Mirau objective alone. Indeed, without the microsphere, the grooves can neither be observed nor measured with the classical interferometric system. The depth of the grooves measured by AFM is 21.0–24.8 nm (Fig. 5d).

**Figure 6:**
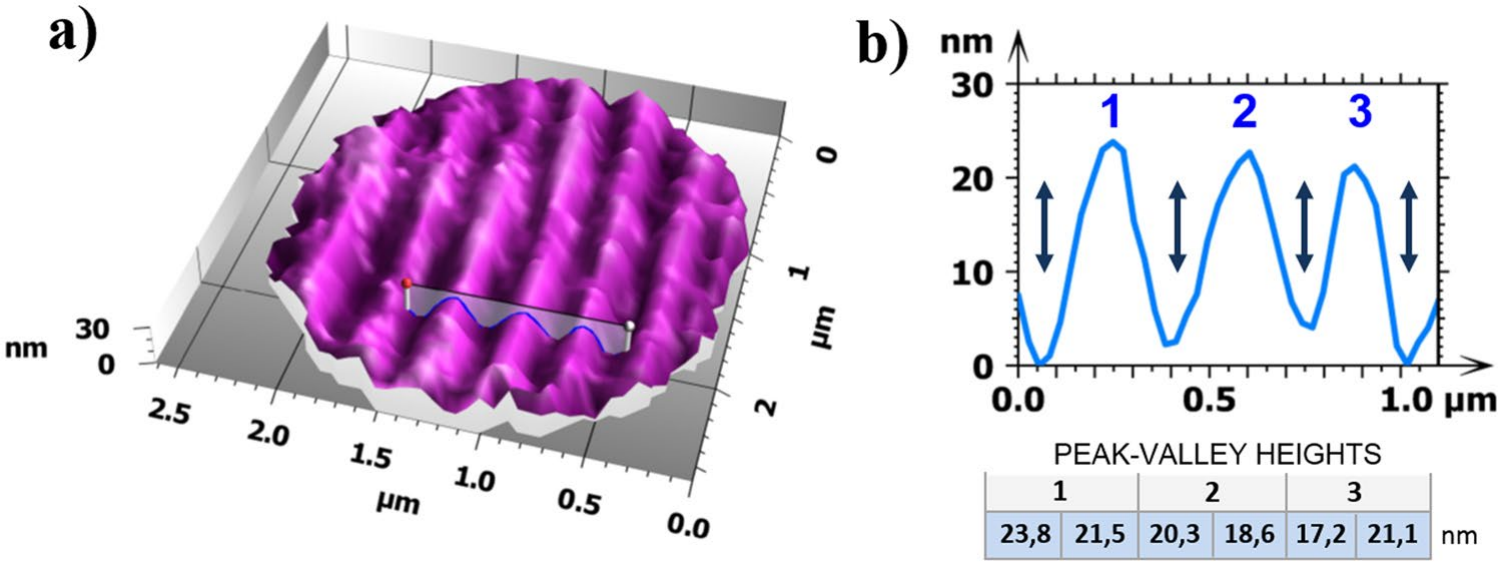
Blu-ray Disc: (**a**) 3D image measured by microsphere assisted interferometry. (**b**) Cross sectional profile from position indicated in (**a**), showing resolved grooves 17.2–23.8 nm deep.

The 3D image after corrections for the aberrations is presented in Fig. 6a. The field of view, of around 2–3 µm, is in good agreement with that reported in the literature of about a quarter the diameter of the microsphere^14, 25^. The depth of the grooves is measured to be between 17.2 nm and 23.8 nm. The general shape of the grooves is near-to-sinusoidal (Fig. 6b), as also found by Wang *et al*.^26^ and confirmed by our own AFM measurements (Fig. 5d). The designed shape of the disc grooves is actually trapezoidal^18^, with well-defined slope angles^23^, as found by experimental measurement^19^. One explanation for the sinusoidal shape being measured by microsphere-assisted interferometry and AFM is that remains from the disc protective layer adhered to the grooves.

## Discussions

The experimental results of microsphere enhanced Mirau interferometry demonstrate a lateral resolution of 112 nm, which is more than five times better than the classical resolution limit of 0.67 µm of the system used and clearly beyond the diffraction limit. Lateral super-resolution has therefore been achieved. Combined with an axial resolution of several nanometers, the ability to improve the lateral resolution of a single-lens far field interferometric microscope using a photonic jet lens considerably extends the performance in terms of imaging and measuring 3D nanometric surface roughness and structures. The simulations confirm the possibility of improving the lateral resolution of 3D measurements compared to classical far-field imaging and suggest that the phase change of the reflected wave plays a major role in nano-resolution imaging.

Finding a suitable polymer sample featuring known X, Y, Z nanometer dimensions was difficult. Having chosen the Blu-ray Disc, a challenge was to remove the cover layer without damaging the layer structure, which could lead to differences between the measured results and the exact design specifications. As indicated previously, for example, residues from the layer removed could affect the measured profile. The slight difference between the heights measured by microsphere assisted interferometry and AFM could be due to the self-assembly procedure not allowing exact positioning of the spheres, or because of slightly different measurement areas being analyzed by each microscope.

While the physics of super-resolution imaging is still unclear, the results of this work have provided some useful contributions to the discussion. For example, the reason why a dielectric microsphere achieves an image in a virtual plane with a magnification larger than one has been explained. Then, a numerical method to predict the position of the virtual plane and the magnification has been proposed. The potential influence of coherence in the resolution improvement has also been pointed out. While the experimental results have verified that the depth of focus of the objective is extended by the photonic jet, they do not fully confirm the exact simulation prediction for the increase in magnification by the photonic jet in the volume of focus (see the single wavelength simulation in Fig. 2b,c). To further explore these exciting results, more realistic simulations need to be performed that take into account the wall angle and the properties of the phase changing material as described by Brusche *et al*.^23^. Such simulations would probably not dramatically change the predictions but could provide a better understanding of the physical phenomena involved in the imaging.

An alternative explanation of the difference in magnification between the results of simulation and experiment may come from considering the interference conditions of the Mirau objective. By adding the microsphere, the optical path length in the sample arm is increased, which unbalances the two arms of the interferometer which normally need to be within the coherence length of the light source (in this case, FWHM = 1.2 µm) to produce interference. In the presence of a planar glass layer in the sample arm, what is known as the coherence gate would be separated from the focal gate, so preventing interference^27^. However, for a small diameter microsphere (11 µm), the two gates still overlap sufficiently along the optical axis since the focal gate is also displaced by the microsphere (in the same direction) and allows the interference fringes to be made visible. Moreover, the experimental results show that the depth of focus of the Mirau objective is increased beyond the value of 0.9 µm in air, possibly due to the effect of the photonic nanojet. This finding supports the notion that the role of the photonic nanojet may be significant. In addition, the fact that the path length difference cannot be modified in the Mirau objective used, means that the optimum focus probably cannot be attained. As a consequence the magnification is only ~x2.6.

During the preparation of this article a microsphere assisted Linnik interferometer system featuring two matched lenses was published^26^, confirming that super-resolution imaging is not confined to Mirau systems. These results may signal the beginning of a new era in quantified 3D surface and sub-surface imaging with applications ranging from malaria screening to the quality assurance of integrated microelectronic circuits.

## Methods

The electromagnetic simulations were performed with a 2D finite element method (FEM) that solves the vectorial propagation equation. TE waves were considered, in which the electric field is orthogonal to the plane studied. The source was introduced as an incident wave on a so-called radiation boundary. Perfectly matched layer absorbing boundary conditions were used for all other boundaries. The space was meshed with a Delaunay triangulation in which the elements are smaller than λ/10. For white light, the spectral range of the source was accounted for by incoherently summing simulations run for wavelengths from 400 nm to 1000 nm in 50 nm steps.

To make the high resolution measurements, the objective was scanned along the optical axis while imaging the sample through the microsphere. The axial scan range was selected to cover all the interference patterns produced by the sphere, limiting the scan to a range of 20 µm. Custom made data acquisition software was used to scan through the range with 68.75 nm steps^28^ taking 8 time averaged pictures for each height position. A high-precision scanner for microscope objectives (PIFOC P-721.CDQ, PI, Germany) was used to ensure precise movement and positioning during the frame grabbing process. From the set of images obtained, the interference data was analysed at each pixel in terms of the light intensity as a function of the relative height. By applying the algorithm from Harasaki *et al*.^28^ an accurate surface position map for every pixel was calculated, so producing a 3D data map. This data was then analysed with commercial software^29^ to extract geometrical values and to produce a 3D view. Since the microsphere adds magnification to that provided by the objective, the lateral scale of the measured 3D data was corrected by using a magnification factor calculated from the ratio between the measured pitch to the nominal pitch retrieved from the literature^18, 19^.

The data acquisition times for scanning through the 20 µm range with 8 time averaged images at each step is a couple of minutes. The scanning time depends on the axial range, the camera frame rate (48 fps in our case), and the averaging. With the system used, for a maximum range of 100 µm, the scanning time is from 20 seconds to a few minutes, depending on the acquisition parameters.

The 323 ± 2.5 nm inter-groove distance (Fig. 5c) was determined from SEM measurements and the associated calibrated values of the pixel size in the SEM data file. The inter-groove distance was calculated by counting the number of pixels between two points and then multiplying this by the pixel size.
